# Multivoxel Pattern Analysis Reveals Auditory Motion Information in MT+ of Both Congenitally Blind and Sighted Individuals

**DOI:** 10.1371/journal.pone.0063198

**Published:** 2013-04-30

**Authors:** Lukas Strnad, Marius V. Peelen, Marina Bedny, Alfonso Caramazza

**Affiliations:** 1 Center for Mind/Brain Sciences, University of Trento, Rovereto, Italy; 2 Department of Psychology, Harvard University, Cambridge, Massachusetts, United States of America; 3 Brain and Cognitive Sciences Department, Massachusetts Institute of Technology, Cambridge, Massachusetts, United States of America; 4 Berenson-Allen Center for Noninvasive Brain Stimulation, Beth Israel Deaconess Medical Center, Harvard Medical School, Boston, Massachusetts, United States of America; University of Cambridge, United Kingdom

## Abstract

Cross-modal plasticity refers to the recruitment of cortical regions involved in the processing of one modality (e.g. vision) for processing other modalities (e.g. audition). The principles determining how and where cross-modal plasticity occurs remain poorly understood. Here, we investigate these principles by testing responses to auditory motion in visual motion area MT+ of congenitally blind and sighted individuals. Replicating previous reports, we find that MT+ as a whole shows a strong and selective responses to auditory motion in congenitally blind but not sighted individuals, suggesting that the emergence of this univariate response depends on experience. Importantly, however, multivoxel pattern analyses showed that MT+ contained information about different auditory motion conditions in both blind and sighted individuals. These results were specific to MT+ and not found in early visual cortex. Basic sensitivity to auditory motion in MT+ is thus experience-independent, which may be a basis for the region's strong cross-modal recruitment in congenital blindness.

## Introduction

In a typically developing human brain, sensory cortices are dominated by inputs from a single modality (e.g. visual cortex, auditory cortex). However, in absence of inputs from the dominant modality during development, strong responsiveness to stimuli from other modalities emerges. This phenomenon – cross-modal plasticity – has been best documented in the visual cortex in blindness. The visual cortex of blind people responds to tactile [1] and auditory stimuli [2], [3]. Furthermore, there is evidence that visual cortex plays a causal role during non-visual tasks [4]. However, the principles determining where and how cross-modal plasticity emerges remain largely unknown. For example, what anatomical changes enable this plasticity? How are the new non-visual functions of visual areas related to their original visual functions? Here, we study these principles in the context of motion processing.

Several recent studies have documented a particularly striking form of visual-to-auditory cross-modal plasticity in the MT+ complex, a motion-selective region in the human visual cortex [5]. Not only does MT+ become responsive to auditory stimuli in the congenitally blind, but it does so in a functionally homologous manner, as indicated by the fact that the MT+ complex in blind people responds preferentially to auditory motion [6]. By studying early-blind sight-recovery individuals in whom MT+ could be functionally localized in both visual and auditory modalities, Saenz and colleagues [7] demonstrated that the middle temporal area that responds to auditory motion in blind individuals indeed corresponds to the functionally defined MT+ complex.

Studies on cross-modal plasticity in MT+ consistently report strong responses to auditory stimuli in blind individuals, but sub- or around-baseline response to auditory stimuli in the sighted. Recently, Bedny and colleagues [8] showed strongly positive responses to auditory motion stimuli in MT+ (as defined in a separate group of sighted participants performing a visual motion task) in congenitally blind individuals but not in late blind or in sighted individuals, suggesting a paramount importance of early sensory experience for this case of cross-modal plasticity. Furthermore, they found that in congenitally blind, but not in late blind or sighted participants, responses in MT+ differentiated between two auditory motion conditions that differed in the degree to which they implied motion: the MT+ of congenitally blind participants responded more strongly to a high motion condition, consisting of footstep sounds increasing or decreasing in volume, relative to a low motion condition, consisting of tones similarly increasing or decreasing in volume but less strongly perceived as motion. No difference between these conditions was found in the MT+ of the late blind and sighted groups.

One interpretation of these findings is that MT+ only differentiates between high and low auditory motion conditions in the absence of developmental visual experience. Alternatively, MT+ may also differentiate between these conditions in the sighted [9] but this may not be reflected in overall response differences in the MT+ complex as a whole. Specifically, it is possible that some voxels of MT+ were activated while other voxels were concurrently deactivated for the high versus low motion contrast in the sighted participants of Bedny et al. [8]. In such a scenario, overall activity would not differentiate between auditory motion conditions, but high and low motion conditions would evoke different activity patterns across the voxels of this region.

To test this possibility, we reanalyzed the data from Bedny et al. [8] using multi-voxel pattern analysis (MVPA). MVPA tests whether spatial patterns of activity differ between conditions. Rather than providing a measure of the overall responsiveness of a region, it provides a measure of the information about experimental conditions contained in multivoxel activity patterns. If MVPA were to reveal information about the high versus low auditory motion conditions in the MT+ of the sighted, this would support the idea that responses to auditory motion in blind individuals develop on the basis of an underdeveloped but inherent ability of MT+ to encode polymodal information about motion [10].

## Methods

### Ethics statement

The study was approved by MIT's Institutional Review Board and written informed consent was obtained from all participants.

### Participants and data

In our analyses, we used the data of congenitally blind and sighted participants from Experiment 1 of Bedny et al. [8]. In total, data from 10 congenitally blind participants, and 20 sighted participants were included. Although a small number of the congenitally blind participants had minimal residual light perception, none of them reported ever having any usable vision (could not see motion, shape, or color or detect objects in their environment, and none of the participants had measurable acuity, see Bedny et al. [8] for details).

### Task and experimental design

During the experiment, participants listened to sound stimuli implying movement. In one condition, the stimuli consisted of human footsteps. In the other condition, they consisted of meaningless tones. The stimuli implied motion in two possible directions – towards or away from the participant. Importantly, the two conditions differed in the amount of motion content implied by the stimuli. Ratings from a separate group of sighted participants established that a stronger percept of motion was associated with the footsteps, compared to the tones. Consequently, we refer to the conditions as high-motion and low-motion, respectively.

In each trial, the task of the participants was to determine the direction (towards or away) of a sound stimulus presented for 2 s at the start of the trial. Therefore, the behavioral task was orthogonal to the amount of motion content of the stimuli. During the fMRI scans, trials were grouped in blocks of 4, with successive stimulus presentations 3 s apart. Within a block, the type of motion (high or low) was kept constant, while the directions were randomized. Individual blocks were separated by 12 s of rest. See Bedny et al. [8] for details and behavioral results.

#### fMRI data processing

Data analysis was performed using AFNI software package [11], PyMVPA package for multivariate analysis [12] and custom-written software in R. For each subject, the high-resolution anatomical image was aligned with the first volume of the first functional run, and subsequently warped into standard Talairach space. The raw time series in each voxel of the functional volumes was time-shifted to account for the temporal order of acquisition of individual slices. The functional volumes were then motion-corrected, and transformed into Talairach space using parameters derived from the warping of the high-resolution anatomical image. All non-brain voxels were masked out from each functional volume, and the time series in each voxel contained within the brain mask was scaled to a common mean. No spatial smoothing was applied to the data used for MVPA. However, we also created a copy of the data that was spatially smoothed with a Gaussian kernel (5 mm full-width half-maximum). This version of the data was used for ROI definition and for univariate analyses.

For univariate analyses, we estimated a general linear model (GLM) for each subject that included a single regressor for each condition. These regressors were created by convolving the boxcar function indicating when each stimulus type block was on with the double-gamma canonical heamodynamic response function. The model further included temporal derivatives of the regressors representing the experimental conditions: six regressors containing the estimated motion-correction parameters to reduce any residual motion-induced signal changes, and constant, linear, quadratic, and cubic dummy regressors for each run to account for signal baseline shifts between runs as well as slow signal drifts within runs. In the univariate ROI analysis beta values were averaged across the voxels of the ROI. For multivariate analyses we estimated a GLM for each subject with the same parameters, but this time modeling each block as a separate regressor.

#### Multivoxel pattern analysis

The classification analyses were performed using a linear support vector machine (SVM) trained and tested on data from each participant in a leave-one-out cross-validation scheme. For each participant, three to four scanning runs were available. In every step of the cross-validation procedure, we withheld one scanning run (each run was withheld once), and trained the SVM on the remaining runs to distinguish between high- and low-motion blocks of trials. In turn, we evaluated the performance of the SVM classifier by computing its accuracy in discriminating between the high- and low-motion conditions in trials from the withheld run. Classification performance values from each step of the cross-validation procedure for each participant were combined by simple averaging. We thus obtained a single classification performance measurement for each participant.

To test whether the classification performance significantly differed from chance at the group level, we used multi-factor ANOVA models. Note that before entering the values into the model, we subtracted the chance level (i.e. 0.5) from all of them so that any difference from zero in an ANOVA would indicate above-chance classification performance.

#### ROI definition

**Figure 1:**
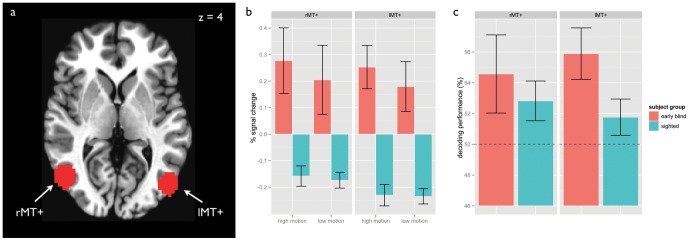
Results of univariate and multivariate analyses. (a) An axial slice of a brain in the standard Talairach space showing a section through the two 10 mm spherical ROIs corresponding to rMT+ and lMT+. (b) Univariate results showing activity in MT+ for high and low motion conditions in congenitally blind and sighted participants. (c) Multivariate results showing classification performance for the decoding of high versus low motion conditions in congenitally blind and sighted participants. The dashed line represents the chance level for classification. Error bars represent the standard error of the mean.

In both sighted and congenitally blind participants, MT+ was based on group peak coordinates from a previous study [13]. In that study, MT+ was functionally defined in a group of sighted participants, and peak coordinates corresponding to lMT+ and rMT+ from the group random-effects model of the localizer were reported (MNI coordinates for rMT+ [48 −66 2], for lMT+ [−46, −72, 3], see also Figure 1a). Our lMT+ and rMT+ ROIs were defined as all voxels within 10 mm radius of these peak coordinates. For multivariate analyses, we reduced the size of the ROIs by applying a feature-selection criterion, including only the 50 voxels with the highest T-values from the contrast task > rest. Control regions BA17 and BA18 were defined using a normalized anatomical atlas [14]. For multivariate analyses, the same voxel selection criterion as for MT+ was applied, again including only the 50 voxels with the highest T-values from the contrast task > rest.

## Results

We first investigated the univariate response to the high motion and low motion conditions in MT+, averaging the beta values across the voxels of the ROI. We computed a three-way ANOVA with subject group, hemisphere, and type of motion as factors (Table 1). This revealed a main effect of group (F[1, 28]  = 27.60, p<0.0001) with MT+ of the blind significantly more active than that of the sighted, and a main effect of hemisphere (F[1, 84]  = 5.89, p = 0.017) with MT+ significantly more active in the left than in the right hemisphere. The intercept, and all other main effects and interactions were not significant. Furthermore, as previously reported (Bedny et al. 2010), there was a significant difference between high and low motion conditions in blind individuals (t(9)  = 2.82, p<0.02) but not in the sighted (t(19)  = 0.47, p = 0.65). Therefore, in line with earlier reports [7], [8], MT+ in the congenitally blind strongly responded to auditory motion stimuli, while the response of MT+ in the sighted was below the rest baseline, and did not differentiate between the two motion conditions (see also Figure 1b).

**Table 1:** Univariate results.

	DF	F-value	p-value
MT+			
(Intercept)	84	2.17	0.145
sub_grp	28	27.60	<.0001
hem	84	5.89	0.017
cond	84	2.04	0.157
sub_grp:hem	84	0.82	0.369
sub_grp:cond	84	1.87	0.176
hem:cond	84	0.03	0.870
sub_grp:hem:cond	84	0.02	0.892

BA17			
(Intercept)	84	0.50	0.480
sub_grp	28	6.09	0.020
hem	84	6.84	0.011
cond	84	0.35	0.557
sub_grp:hem	84	7.49	0.008
sub_grp:cond	84	0.00	0.966
hem:cond	84	0.02	0.892
sub_grp:hem:cond	84	0.01	0.927

BA18			
(Intercept)	84	3.31	0.073
sub_grp	28	7.49	0.011
hem	84	1.87	0.175
cond	84	0.75	0.388
sub_grp:hem	84	0.04	0.837
sub_grp:cond	84	0.09	0.766
hem:cond	84	0.02	0.888
sub_grp:hem:cond	84	0.06	0.805

Factor legend:

sub_grp  =  subject group.

hem  =  hemisphere.

cond  =  condition.

In order to test whether MT+ of the sighted and of the congenitally blind is nevertheless sensitive to auditory motion information, we used multi-voxel pattern analysis. We trained a support vector machine (SVM) to discriminate between patterns associated with high motion and low motion auditory stimuli. Sensitivity to auditory motion information in a region would be indexed by a classification performance significantly different from chance (i.e. 0.5). Before training, multi-voxel patterns were normalized to mean zero in each condition. This was done so that the classifier could not use information about differences in the overall mean activation in a region to discriminate between conditions (i.e. the same information that univariate analyses are based on), and instead rely only on distributed patterns of activation.

The classification performance in left MT+ (lMT+) and right MT+ (rMT+) for both groups of subjects is displayed in Figure 1c. To determine whether the performance was above chance in either group, we computed a two-way ANOVA with subject group and hemisphere included as factors (Table 2). The ANOVA model yielded an intercept significantly greater than 0.5 (F[1, 28]  = 15.52, p  = 0.0005), but no significant effect of subject group or hemisphere (F[1, 28]  = 2.78 p = 0.11). In other words, the decoding performance is above chance in both groups (both F>5.9, and p<0.03), and does not differ significantly between the two groups. This suggests that information about auditory motion is present in MT+ in the sighted as well as blind individuals.

**Table 2:** MVPA results.

	DF	F-value	p-value
MT+			
(Intercept)	28	15.52	0.001
sub_grp	28	2.78	0.107
hem	28	0.04	0.852
sub_grp:hem	28	0.63	0.434

BA17			
(Intercept)	28	2.61	0.118
sub_grp	28	1.42	0.243
hem	28	0.00	0.952
sub_grp:hem	28	0.00	0.949

BA18			
(Intercept)	28	1.66	0.208
sub_grp	28	0.72	0.403
hem	28	3.61	0.068
sub_grp:hem	28	0.31	0.584

Factor legend:

sub_grp  =  subject group.

hem  =  hemisphere.

Even though the main effect of hemisphere was not significant in the multivariate analyses, we do report hemispheric differences in MT+ in the univariate analyses, consistent with Bedny et al. [8]. Therefore, we also tested for differences in decoding performance between the two groups separately in rMT+ and lMT+. We found that classification performance was marginally better in the congenitally blind group in lMT+ but not in rMT+ (lMT+: t(28) = 2.02, p = .05, rMT+: t(28) =  0.69, p = 0.50).

In order to verify that the effects reported above are specific to MT+, we also performed MVPA in anatomically defined control regions: Brodmann Area 17 (approximately corresponding to visual area V1) and Brodmann Area 18 (approximately corresponding to V2 and V3). Classification performance was not above chance in these control regions in either group (Table 2).

## Discussion

We have shown that MT+ is sensitive to auditory motion information independently of visual experience and despite large differences in overall responsiveness of the region in the sighted and the early blind. While univariate analyses revealed strong and selective activity for auditory motion in MT+ in blind individuals but not in the sighted, multivariate analyses revealed that activity patterns in this region differentiate between different auditory motion conditions in both groups. Sensitivity to auditory motion conditions in MT+ is thus experience-independent, which may be a basis for the region's strong cross-modal recruitment in congenital blindness.

An outstanding question concerns what neural responses in MT+ lead to MVPA decoding of auditory motion. One possibility is that some parts of the MT+ complex were activated by auditory motion, while other parts were deactivated. As an example of such a scenario, a recent study [15] showed that in sighted individuals tactile motion activates the anterior part of MT+ (MST) and concurrently deactivates the posterior part of MT+ (MT). By contrast, the same contrast activated the full MT+ of congenitally blind individuals. In our study, whole-brain group analyses revealed no evidence for a clear anterior-posterior activity difference for the high versus low motion contrast in the sighted group, even at low statistical thresholds. This suggests that the activity patterns that differentiated between the high and low motion conditions in our study were relatively distributed across the region and/or did not spatially overlap across individuals.

Our results suggest that information about the degree of motion inferred from auditory input is present in MT+ in both blind and sighted people (see [16], [17] for evidence for above-chance decoding of direction of auditory motion in MT+ in blind [17] but not in sighted [16]). Studies with sight recovery individuals further suggest that auditory and visual motion responses can coexist in MT+. Saenz and colleagues [7] reported two cases of early blind patients whose sight was restored in adulthood. After regaining sight, these individuals retained auditory motion responses in MT+, but the region also started responding selectively to visual motion as it does in sighted individuals. Therefore, development of strong responses to auditory motion in MT+ does not prevent neural circuits from processing visual motion. We therefore hypothesize that in the absence of visual experience cross-modal plasticity in MT+ takes place through strengthening of existing non-visual inputs, which are typically weak relative to the visual inputs [10]. The same pathways that carry auditory motion to MT+ in sighted people likely come to drive the response of this area in blind individuals.

A possible objection to our interpretation is that the presence of information about auditory motion in MT+ of the sighted is a result of visual imagery. While we cannot rule this out conclusively, there are good reasons to think that visual imagery cannot account for the present pattern of results in the sighted. First, visual imagery cannot explain the multivoxel information about auditory motion in MT+ of congenitally blind individuals, as they have never seen, although it remains possible that the presence of information about motion is mediated by visual imagery in the sighted and by a different process in blind individuals. Second, previous research has shown that visual imagery of motion increases overall activity in MT+ [18], while no such difference in activity was observed here.

Following Bedny et al. [8], we interpret the univariate (early blind) and multivariate (early blind and sighted) differences between the two motion conditions in MT+ as reflecting differences in the motion properties of the stimuli; an independent group rated the footsteps condition as implying more motion than the tones condition. However, the two conditions also differed in other respects, including low-level sound properties (see [8]). Furthermore, footsteps imply the presence of a walking person, possibly recruiting nearby regions involved in body and/or biological motion processing [19], [20]. Therefore, although our results in MT+ likely reflect differences in the motion properties of the stimuli, it cannot be excluded that univariate or multivariate differences between the high- and low-motion conditions additionally reflected other differences between these conditions.

Our findings are closely related to several recent studies highlighting instances of cross-modal plasticity that broadly preserve the original function of the affected region. We have focused on an example from the domain of motion processing. Related studies have found that dorsal stream regions involved in visuospatial processing in sighted individuals are involved in auditory-spatial processing in congenitally blind individuals [21], [22]. In another study, Renier and colleagues [2] investigate the middle occipital gyrus (MOG), an area known to be involved in visuo-spatial processing in the sighted. They found that in blind individuals, this area develops sensitivity for spatial processing in the auditory and haptic modalities, again suggesting that in some cases cross-modal plasticity is predicted by the broad functional characteristics of the area even as the nature of sensory inputs driving it changes dramatically. Other studies include studies of reading and the visual word form area (VWFA). In the sighted, this region selectively responds during visual reading [23]. The authors reported that in the early blind people, an area anatomically corresponding to VWFA becomes active during Braille reading. Finally, Mahon and colleagues [24] showed that the large-scale organization of object representations by category in the ventral stream exists independently of visual experience (see also [25], [26]). Taken together, all of the above studies exhibit the same intriguing pattern across diverse brain regions: selectivity for stimuli within a cognitive domain is preserved in the face of categorically different sensory inputs and altered responses across sensory-modalities.

The co-location of information about visual and auditory motion in MT+ has broad theoretical implications. The fact that a single region contains diverse information about a specific domain, and that such configuration is invariant to sensory experience highlights the fact that plasticity is constrained by the functional architecture of the brain. Such constraints may come in various forms. For example, according to the metamodal-theory of brain function [10], functionally distinct brain areas, including many of those traditionally thought of as unimodal sensory areas, are uniquely characterized by the types of computations they perform, independently of the modality of inputs over which they operate. On this account the internal circuitry of MT+ determines its predisposition for motion processing. Cross-modal plasticity could then be seen as acting to modulate the relative importance of inputs from each modality in any given area, rather than qualitatively changing the nature of the underlying computations. At the same time, the kind of information that is encoded in a brain region may be constrained by the pattern of connectivity of the region to other areas [27]. Even when functional selectivity is preserved across modalities, cross-modal responses may nonetheless reflect distinct underlying computations. In the case of MT+, computations over auditory input in blind individuals could be qualitatively different from computations over visual input in the sighted. While our data cannot adjudicate between these and other similar principles, they suggest that cross-modal plasticity may be guided by preexisting constraints on brain organization.
